# Factors influencing choice of surgical route of repair of genitourinary fistula, and the influence of route of repair on surgical outcomes: findings from a prospective cohort study

**DOI:** 10.1111/j.1471-0528.2012.03461.x

**Published:** 2012-08-20

**Authors:** V Frajzyngier, J Ruminjo, F Asiimwe, TH Barry, A Bello, D Danladi, SO Ganda, S Idris, M Inoussa, M Lynch, F Mussell, DC Podder, MA Barone

**Affiliations:** a EngenderHealthNew York, NY, USA; b Kagando Mission HospitalKagando, Kasese District, Uganda; c Prefectoral Hospital of KissidougouKissidougou, Guinea; d Maryam Abacha VVF Centre and Women and Children HospitalSokoto State, Nigeria; e Specialist Fistula CentreBirnin Kebbi, Kebbi State, Nigeria; f Lamordé HospitalNiamey, Niger; g Faridat Yakubu HospitalZamfara State, Nigeria; h Maradi HospitalMaradi, Niger; i Kitovu Mission HospitalMasaka, Uganda; j LAMB HospitalParbatipur, Bangladesh; k Kumudini HospitalNarayanganj, Bangladesh

**Keywords:** Developing countries, genitourinary fistula, observational study, propensity score, route of repair, surgery

## Abstract

**Objective:**

The abdominal route of genitourinary fistula repair may be associated with longer term hospitalisation, hospital-associated infection and increased resource requirements. We examined: (1) the factors influencing the route of repair; (2) the influence of the route of repair on fistula closure 3 months following surgery; and (3) whether the influence of the route of repair on repair outcome varied by whether or not women met the published indications for abdominal repair.

**Design:**

Prospective cohort study.

**Setting:**

Eleven health facilities in sub-Saharan Africa and Asia.

**Population:**

The 1274 women with genitourinary fistula presenting for surgical repair services.

**Methods:**

Risk ratios (RRs) and 95% confidence intervals (95% CIs) were generated using log-binomial and Poisson (log-link) regression. Multivariable regression and propensity score matching were employed to adjust for confounding.

**Main outcome measures:**

Abdominal route of repair and fistula closure at 3 months following fistula repair surgery.

**Results:**

Published indications for abdominal route of repair (extensive scarring or tissue loss, genital infibulation, ureteric involvement, trigonal, supratrigonal, vesico-uterine or intracervical location or other abdominal pathology) predicted the abdominal route [adjusted risk ratio (ARR), 15.56; 95% CI, 2.12–114.00]. A vaginal route of repair was associated with increased risk of failed closure (ARR, 1.41; 95% CI, 1.05–1.88); stratified analyses suggested elevated risk among women meeting indications for the abdominal route.

**Conclusions:**

Additional studies powered to test effect modification hypotheses are warranted to confirm whether the abdominal route of repair is beneficial for certain women.

## Introduction

Genitourinary fistula is predominantly a childbirth-associated morbidity, whereby prolonged pressure of the fetus’s head during obstructed labour results in an abnormal passage between the vagina and bladder or between the vagina and rectum, resulting in urinary or faecal incontinence, or both. Fistulas resulting in urinary incontinence are most common, and are often referred to as genitourinary fistulas. Although the majority (80–95%) of genitourinary fistulas can be closed surgically,^1^ the likelihood of successful closure depends on the characteristics and severity of the fistula, skill of the surgeon and, probably, the surgical methods used. Many fistula surgeons have developed their own methods through experience^2^; thus, perioperative procedures vary widely across surgeons and facilities. Few studies have examined the comparative effectiveness of different perioperative interventions related to the surgical management of genitourinary fistulas.^3^–^13^ One aspect of surgical repair in particular, the route of repair undertaken, is of critical research interest, as the abdominal (versus vaginal) approach may be associated with longer term hospitalisation,^14^ urinary tract infection (UTI)^15^ and increased blood loss.^14^,^15^

Recommendations vary with regard to whether a vaginal or abdominal surgical approach should be used for fistula repair. Vaginal approaches are generally thought to be appropriate for any fistula located between the bladder and the vagina,^16^,^17^ with some fistula surgeons claiming to be able to repair all fistulas by the vaginal route.^18^ However, abdominal approaches are also often considered to be most appropriate for ‘complex’ fistulas,^14^,^19^,^20^ with published indications for an abdominal route of repair including: a small-capacity or poorly compliant bladder which requires bladder augmentation^14^,^17^,^19^; fistulas involving or close to the ureteric orifice (particularly if ureteric reimplantation is required)^14^,^17^,^19^; vaginal stenosis or other factors inhibiting adequate vaginal exposure of the fistula^14^,^17^,^19^; size^14^; trigonal or supratrigonal location^14^; intracervical location^18^; and concomitant abdominal pathology.^19^ However, the choice of surgical approach remains, to some extent, a matter of surgeon preference or training^19^,^21^ and experience of the surgical team.^14^

Three retrospective studies have examined unadjusted associations between the route of surgery and repair outcomes. Kriplani et al.^9^ found a significantly higher proportion of continence (closed fistula with no residual incontinence) at discharge among fistulas repaired vaginally in their sample of 34 women. In contrast, Chigbu et al.,^5^ in their sample of 78 women with juxtacervical fistulas (which can be approached either vaginally or abdominally^5^), found a higher proportion of fistula closure at discharge among women repaired abdominally (84.3%) than vaginally (77.8%); however, this difference was not statistically significant. Finally, Morhason-Bello et al.^15^ found no statistically significant differences in continence across 71 women with mid-vaginal fistula (with no fibrosis or evidence of infection, urethral or bladder neck involvement and without more than one previous repair) repaired either abdominally or vaginally; continence rates 3 months following surgery were 78.3% versus 80.0%, respectively. All three studies were probably underpowered to detect small differences, and examined only unadjusted associations (although the last two studies restricted the sample by type of fistula). Only Morhason-Bello et al. examined indications for vaginal versus abdominal or mixed vaginal and abdominal route of repair; the number of indications examined was limited because of the strict inclusion criteria employed.

A shared limitation of all three studies was the lack of adjustment for the potential imbalance of a range of prognostic features across comparison groups, also termed ‘confounding by indication’. In an observational study, the indication for a treatment may act as a confounder.^22^ For instance, a patient’s urinary fistula may have certain characteristics which indicate the need for an abdominal route of repair and, at the same time, these characteristics may also be associated with a poor repair prognosis. Consequently, treatments reserved for those with a poor prognosis will be statistically associated with worse outcomes, even when the treatment itself is beneficial.^23^ Although observational studies typically rely on methods such as statistical adjustment to minimise differences between comparison groups, confounding by indication may be less amenable to standard ways of accounting for confounding.^23^ For example, methods of controlling for noncomparability of comparison groups, such as disease severity scores, may not encompass the totality of factors that may influence both a provider’s decision with regard to the route of repair and eventual repair outcomes. This would result in incomplete adjustment and residual confounding.

Propensity score matching has been proposed as a method particularly suited for the control of confounding by indication. These methods are used to approximate the context of a randomised trial, insofar as treatment groups are comparable on measured confounding factors. Propensity score matching may thus minimise selection bias, as it maximises the comparability of individuals on a set of observed variables that may influence the provider’s decision to administer the treatment: in this case, route of repair.^24^ Importantly, however, propensity score matching cannot ensure comparability on unmeasured confounding factors.

Against this background, we conducted a secondary analysis on data from a multi-country prospective cohort study to elucidate the relationship between the route of repair and fistula closure. Our first aim was to evaluate which factors independently predicted the route of repair used, including the extent to which the choice of abdominal route was influenced by published indications for an abdominal route of repair. Second, we aimed to examine the influence of the route of repair on fistula closure, using both standard multivariable regression analysis and propensity score matching to account for potential confounding. Our third aim was to evaluate whether the effect of the route of repair on fistula closure varied by indication for repair.

## Methods

### Study participants

Between September 2007 and September 2010, 1389 women presenting for fistula repair services at 11 study sites in five countries (Uganda, Guinea, Niger, Nigeria, Bangladesh) were enrolled in the study, 1329 of whom underwent urinary fistula repair. Of the women who did not undergo urinary fistula repair, 25 underwent repair for rectovaginal fistula only (and were therefore excluded from these analyses) and 35 women were referred to other facilities, did not have surgery for medical/safety reasons or were treated by catheterisation; these women were evenly distributed across all facilities. Retention was high, with 95.9% of women returning for a 3-month follow-up visit; the 1274 women retained constituted the study sample for these analyses. The median duration of follow-up was 88.0 days [interquartile range (IQR), 84.0–99.0 days]. All women provided signed informed consent (if the woman was not literate, consent was indicated via thumbprint).

### Study procedures

Prior to surgery, women reported their sociodemographic characteristics and obstetric history. Information was also collected on co-morbidities and any medical care provided for these co-morbidities. At the time of surgery, detailed information was collected on the characteristics of the fistula, the intraoperative procedures performed and surgical outcomes. Following the surgery but prior to discharge, data were collected on the post-operative care provided, and, at discharge, information was once again collected on surgical outcomes. Women were provided with an appointment to return 3 months following surgery and, when contact information was available, were reminded to return prior to the 3-month mark. A clinical evaluation was conducted at the 3-month follow-up visit to assess surgical outcomes. Women returning at the 3-month visit were reimbursed for their travel expenses.

### Measures

Our first aim was to evaluate which factors independently predicted the route of repair. The primary outcome measure for this aim was the surgical route. Three possible surgical routes can be used: vaginal, abdominal or combined. As we were interested in the abdominal route of repair, irrespective of whether it was used in combination with a vaginal approach, and as analyses (not presented) excluding individuals undergoing combined route of repair generated similar results, we dichotomised route as either ‘abdominal/combined abdominal and vaginal’ (hereafter referred to as ‘abdominal’) or ‘vaginal’.

The potential predictors of an abdominal route of repair included patient characteristics, fistula characteristics, surgeon experience and study site. The patient characteristics assessed included age (reference, 25 years or less), years living with the fistula, marital status (currently married versus unmarried), rural residence, education (reference, less than primary education), parity (reference, three or less) and whether or not the patient had previously undergone surgery to repair the fistula. The co-morbidities assessed included malnutrition (as determined through skin-fold measurement, body mass index or visual assessment), anaemia (as determined through haemoglobin level, haematocrit or visual assessment), UTI (measured using clinician report), urine-induced contact dermatitis, fever, foot drop and type of female genital cutting present, if any.

The fistula characteristics assessed included bladder size, fistula size and location. Bladder size was dichotomised as small versus normal or distended (as defined subjectively by the surgeon), and fistula size was dichotomised at 4 cm or greater. A composite variable representing ureteric involvement was created, and defined as ureteric or uretero-vaginal location, or if ureters were described to be draining into the vagina or at the edge of the fistula. Urethral involvement was categorised as ‘partial’ (urethra involved but not completely destroyed or transected) or ‘complete destruction or transection’. Fistula locations assessed included vesico-uterine, mid-vaginal, juxtacervical, intracervical, trigonal, supratrigonal and vault. Based on the published indications for the abdominal route of repair and the factors plausibly indicative of limited vaginal access and significantly associated with an abdominal route of repair in our data, we created a composite variable representing ‘abdominal repair indicated’. Specifically, this variable comprised the following indications: the presence of extensive scarring or tissue loss, ureteric involvement, trigonal, supratrigonal, intracervical or vesico-uterine location, concomitant abdominal pathology and female genital infibulation.

Surgeon experience was measured by the number of complex repairs conducted by the surgeon; complex was defined subjectively by surgeons, and the variable was dichotomised at 200 complex repairs or greater. Variables related to the context of the repair included whether the repair was conducted as part of a training session or whether it was conducted as part of an outreach or ‘camp’ setting by a team of visiting surgeons.

Our second aim was to examine the influence of the route of repair on fistula closure. The primary exposure measure was the route of repair, as described above. The primary outcome measure was fistula closure 3 months following the surgery, whereby the fistula was characterised dichotomously as either ‘closed’ or ‘not closed’. The main mechanism of assessment of fistula closure was through a pelvic examination and dye test that were routine at nine of the sites. At the two sites (186 women, or 14.6% of cases) at which pelvic examinations were not routinely conducted at follow-up, fistula closure at the 3-month follow-up visit was determined using the question, ‘does the client have continuous and uncontrolled leakage of urine’; this question has been used in household-based Demographic and Health Surveys^25^ to differentiate between fistula and other forms of incontinence, which are unlikely to be continuous and uncontrolled. Any woman complaining of urine leakage at these sites underwent a pelvic examination. In the event that a woman had multiple fistulas, closure refers to closure of all fistulas. For two women with multiple fistulas, the surgery represented the first of a staged repair, and their fistulas were thus considered ‘closed’ despite continued leakage from the remaining fistulas. Potential confounding variables eligible for inclusion in each model were those factors associated with the procedures in question, as well as fistula closure.

The third aim assessed whether the relationship between the route of repair and fistula closure was modified by the variable indication for an abdominal approach, described above.

All continuous variables that did not have a linear effect with respect to the outcome were categorised in a manner that preserved parsimony and ensured homogeneity across strata; these variables included age, parity, fistula size and provider experience in conducting complex repairs, as discussed above.

### Statistical analyses

#### Bivariable analyses

The unadjusted effects of patient and fistula characteristics and provider experience on the route of repair were evaluated using RRs and corresponding 95% confidence intervals (95% CIs); these were generated using the log-link function and binomial distribution specification in SAS PROC GENMOD.^26^ Generalised estimating equations (GEEs) were used to account for the clustering of patient outcomes within facilities.

The characteristics of women whose fistulas were closed at the 3-month follow-up visit were compared with those whose fistulas were not closed using RRs and corresponding 95% CIs; these were similarly derived using GEEs.

#### Multivariable analyses

We first assessed the independent predictors of an abdominal route of repair using multivariable GEE models with the log-Poisson specification and robust variance in GENMOD (SAS PROC GENMOD’s Poisson regression capability was used to facilitate model convergence).^27^ Variables eligible for inclusion in the models were conceptually associated with the procedure, as well as statistically associated (*P* < 0.20) with the procedure in bivariable analysis. In the event that variables were too highly correlated, only one was included. Thus, parity was included rather than age, as increased parity could be correlated with increased pelvic tissue laxity/flexibility, which may influence the choice of surgical approach. Fever, foot drop and malnutrition were not included in the model because of sparse cell sizes.

In order to examine the effect of the route of repair on repair outcome (i.e. failure to close the fistula), we similarly created multivariable GEE models using the log-Poisson specification in GENMOD. These models adjusted for factors conceptually associated with both the route of repair and outcome, as well as statistically associated (*P* < 0.20) with both variables in bivariable analysis.

For our third aim, we conducted stratified analyses to visually assess trends in effect sizes across levels of the potential effect modifier; the presence of multiplicative interaction could not be tested statistically because of insufficient power. Analyses of the effect of the route of repair on fistula closure were stratified by our measure ‘abdominal approach indicated’. Bivariable GEE models were used to generate unadjusted RRs and corresponding 95% CIs for these stratified analyses.

#### Propensity score analysis

Predicted probabilities (propensity scores) of an abdominal route of repair were estimated using two separate multivariable logistic regression models. These propensity score models were developed iteratively, until an optimal balance on the measured covariates was achieved. The first model included a reduced set of variables: abdominal route of repair indicated, mid-vaginal fistula, juxtacervical fistula, partial urethral damage and complete urethral damage/transection. The second model included the same measures, in addition to surgeon experience, site and parity greater than three, in order to improve balance in covariates across groups. Probabilities of abdominal/mixed vaginal and abdominal repair were calculated. Matching was performed using a 1:2 ratio, an optimised matching algorithm and an absolute difference in propensity score of 0.1. Exposed individuals for whom no suitable unexposed match could be found were excluded from the analysis. Eleven women undergoing an abdominal route of repair (19%) were excluded using the reduced propensity score model, and 27 (57%) were excluded with the expanded model. All statistical analyses were conducted using SAS version 9.2 (SAS Institute, Inc., Cary, NC, USA), and statistical significance was two-sided at *P* < 0.05 unless stated otherwise.

## Results

### Patient characteristics

Patient characteristics are shown in Table 1. The women included had a median age of 25 years (IQR, 20–35 years) and a median parity of two (IQR, 1–5). Over one-half (65.1%) of the women were currently married, although over one-quarter (27.1%, not shown) were divorced or separated from their husbands. The majority of women were from rural areas, and one-fifth had at least a primary education. Almost three-quarters of the women with fistulas of obstetric origin had laboured at home for more than 24 hours, and over one-third ultimately delivered via caesarean section. The median number of years that women had lived with their fistula was 3.3, and almost one-quarter of women had previously undergone surgery to repair their fistula. One-fifth of the women presented had undergone female genital cutting, the majority of these being Type II (excision of the clitoris with partial or total removal of the labia minora) or III (genital infibulation). The proportion of women whose fistulas were closed at follow-up was 81.6%.

**Table 1:** Patient characteristics

Patient characteristic	*n* (%)
**Total**	1273
**Median parity (IQR)**	2 (1–5)
**Median age, years (IQR)**	25 (20–35)
**Currently married**	830 (65.1)
**Rural residence**	1088 (86.1)
**>Primary education**	265 (20.8)
**Laboured at home >24 hours during causative delivery**	614 (72.7)
**Delivered via caesarean section during causative delivery**	481 (38.9)
**Years with fistula, mean (SD)**	3.3 (5.5)
**Previously repaired**	295 (23.2)
Female genital cutting*	
None	1012 (79.6)
Type I	33 (2.6)
Type II	124 (9.8)
Type III	97 (7.6)
Other	5 (0.4)
**Co-morbidities**	
Malnutrition	76 (6.0)
Anaemia	91 (7.1)
Fever	21 (4.6)
Uterine tract infection	2 (0.2)
Foot drop	64 (5.0)
**Commodities and utilities in household**	
Piped water	288 (22.7)
Flush toilet	46 (3.6)
Electricity	256 (20.1)
Radio	881 (69.2)
TV	199 (15.7)
Mobile phone	457 (36.0)
Landline phone	24 (1.9)
Refrigerator	49 (3.9)
**Met indications for abdominal route of repair**	400 (31.7)
**Surgical approach**	
Vaginal	1216 (95.52)
Abdominal	47 (3.69)
Mixed	10 (0.79)
**Surgical outcomes**	
Fistula closed at discharge	1058 (84.7)
Fistula closed at 3-month visit	1039 (81.6)

IQR, interquartile range; SD, standard deviation.

* Type I, excision of prepuce, with or without excision of clitoris, or part of clitoris; Type II, excision of the clitoris with partial or total removal of the labia minora; Type III, excision of part of all or the external genitalia and narrowing of the vaginal opening.

### Predictors of route of repair

An abdominal route of repair was used in only 57 of 1273 (5%) women; information on the route of repair was missing for one woman. The use of a vaginal versus abdominal route of repair differed by both facility- and individual-level factors (Tables 2 and 3). Abdominal repairs were more likely to be conducted at four of the facilities, and were never conducted at four others. The likelihood of abdominal repair was inversely associated with surgeon experience in conducting complex repairs [adjusted risk ratio (ARR), 0.49; 95% CI, 0.25–0.95); however, the latter variable was missing for 64 women (repaired by nine of 51 attending surgeons). Women with a parity of three or more (ARR, 1.80; 95% CI, 1.31–2.46) were significantly more likely to undergo an abdominal route of repair. Women with fistulas meeting indications for abdominal repair had a greater than 15-fold higher likelihood (ARR, 15.56; 95% CI, 2.12–114.00) of having an abdominal repair. Fistulas that were mid-vaginal were significantly less likely to be repaired abdominally (ARR, 0.19; 95% CI, 0.05–0.80), and fistulas involving the urethra, either partially or completely, were less likely to be repaired abdominally (partial: ARR, 0.35; 95% CI, 0.15–0.82; completely: ARR, 0.31; 95% CI, 0.13–0.74). Previous repair and the presence of a juxtacervical fistula did not independently predict an abdominal route of repair.

**Table 2:** Patient, fistula and contextual factors by route of repair undertaken

	Abdominal/combined, *n* (%)	Vaginal, *n* (%)
**Total (*n* = 1273)**	57	1216
**Patient characteristics at baseline**		
Parity > 3	34 (61.8)	410 (34.9)
Age > 25 years	41 (71.9)	566 (46.5)
Currently married	36 (63.2)	793 (65.2)
Rural residence	52 (91.2)	1035 (85.8)
>Primary education	19 (33.3)	246 (20.3)
Average years with fistula (SD)	4.1 (6.5)	3.2 (5.4)
Malnutrition	3 (5.3)	72 (5.9)
Anaemia	6 (10.5)	84 (6.9)
Fever	0 (0.0)	20 (4.7)
Uterine tract infection	0 (0.0)	2 (0.2)
Foot drop	0 (0.0)	64 (5.3)
Female genital infibulation	11 (9.3)	40 (7.8)
Previous repair	14 (24.6)	281 (23.2)
**Fistula characteristics**		
Abdominal repair indicated*	52 (91.2)	447 (37.2)
Fistula size ≥ 4 cm	8 (15.7)	248 (21.3)
Small bladder	12 (23.5)	326 (28.8)
Extensive scarring	2 (3.5)	93 (7.7)
Extensive tissue loss	8 (15.4)	127 (10.5)
Extent of urethral damage		
No damage	48 (87.3)	710 (58.5)
Partial damage	3 (5.5)	278 (22.9)
Complete transection/destruction	4 (7.4)	222 (18.4)
Mid-vaginal location	3 (5.4)	366 (30.2)
Trigonal location	6 (10.5)	60 (5.0)
Supratrigonal location	7 (12.3)	25 (2.1)
Juxtacervical location	5 (8.9)	219 (18.2)
Intracervical location	7 (12.5)	74 (6.1)
Vesico-uterine location	10 (17.9)	11 (0.9)
Vault location	3 (5.3)	32 (2.7)
Ureter involvement	25 (43.9)	183 (15.2)
Other abdominal pathology	1 (1.8)	1 (0.1)
**Facility level factors/characteristics**		
Site A	9 (15.8)	61 (5.0)
Site B	2 (3.5)	46 (3.8)
Site C	0 (0.0)	5 (0.4)
Site D	8 (14.0)	238 (19.6)
Site E	0 (0.0)	67 (5.5)
Site F	0 (0.0)	68 (5.6)
Site G	1 (1.8)	52 (4.3)
Site H	1 (1.8)	207 (17.0)
Site I	0 (0.0)	146 (12.0)
Site J	26 (45.6)	133 (10.9)
Site K	10 (17.5)	193 (15.9)
**Surgeon performed over 200 complex repairs**	7 (12.3)	404 (35.0)

SD, standard deviation.

* Female genital infibulation, extensive scarring, extensive tissue loss, trigonal, supratrigonal, intracervical, vesico-uterine location, ureter involvement or concomitant abdominal pathology.

**Table 3:** Unadjusted and adjusted relative risks (RRs) for abdominal versus vaginal (reference) route of repair

	Unadjusted RR (95% CI)	Adjusted RR (95% CI)
**Patient characteristics at baseline**		
Parity > 3	2.88 (1.33–6.20)**	1.80 (1.31–2.46)**
Age > 25 years	2.48 (1.40–4.39)**	–***
Currently married	1.19 (0.61–2.32)	
Rural residence	1.05 (0.66–1.69)	
>Primary education	1.30 (0.86–1.97)	
Average years with fistula (SD)	1.02 (0.97–1.07)	
Malnutrition	0.41 (0.30–0.57)**	–****
Anaemia	1.19 (0.35–4.02)	
Female genital infibulation	2.42 (1.69–3.47)**	
Previous repair	1.29 (1.03–1.62)**	1.06 (0.77–1.47)
**Fistula characteristics**		
Abdominal repair indicated*****	15.76 (2.34–106.06)**	15.56 (2.12-114.00)**
Fistula size ≥ 4 cm	0.76 (0.34–1.69)	
Small bladder	1.10 (0.57–2.11)	
Extensive scarring	0.40 (0.06–2.57)	
Extensive tissue loss	1.68 (0.69–4.08)	
Extent of urethral damage		
No damage	Reference	Reference
Partial damage	0.19 (0.03–1.13)*	0.35 (0.15–0.82)**
Complete transection/destruction	0.38 (0.15–1.00)*	0.31 (0.13–0.74)**
Mid-vaginal location	0.11 (0.02–0.75)**	0.19 (0.05–0.80)**
Trigonal location	2.20 (0.61–7.95)*	
Supratrigonal location	4. 91 (0.97–24.85)*	
Juxtacervical location	0.45 (0.13–1.48)*	0.53 (0.19–1.47)
Intracervical location	1.72 (0.61–4.80)	
Vesico-uterine location	9.78 (4.91–19.44)**	
Vault location	1.15 (0.29–4.51)	
Ureter involvement	4.07 (1.86–8.92)**	
Other abdominal pathology	6.33 (3.72–10.77)**	
**Facility level factors/characteristics**		
Surgeon performed over 200 complex repairs	0.34 (0.24–0.48)**	0.49 (0.25–0.95)**

CI, confidence interval; SD, standard deviation.

* *P* < 0.20.

** *P* < 0.05.

*** Not included in multivariate model because of correlation with parity.

**** Not included in multivariate model because of sparse cell sizes.

***** Includes female genital infibulation, extensive scarring, extensive tissue loss, trigonal, supratrigonal, intracervical, vesico-uterine location, ureter involvement or concomitant abdominal pathology; these variables were therefore not included separately in multivariate models.

### Influence of route of repair on fistula closure

Almost one-fifth (18.8%) of those repaired vaginally experienced repair failure, compared with 10.5% of those repaired abdominally. In bivariable analysis, a vaginal route of repair was associated with 1.42 (95% CI, 1.11–1.81) times the risk of failure to close the fistula relative to the abdominal route. After adjusting for indication for abdominal route of repair, surgeon experience in conducting complex repairs, mid-vaginal location, and partial and complete urethral involvement (factors associated with both routes of repair discussed above, as well as fistula closure), the likelihood of a vaginal route of repair relative to an abdominal route remained similar (ARR, 1.41; 95% CI, 1.05–1.88). Analyses conducted in the propensity score-matched sample, in which propensity scores were created using a reduced set of predictors, found a stronger magnitude of effect relative to the fully adjusted multivariable model (ARR, 1.98; 95% CI, 1.27–3.07); analyses conducted using the expanded propensity score model found an effect similar to that of the fully adjusted multivariable model (ARR, 1.40; 95% CI, 0.77–2.56; Table 4).

**Table 4:** Relative risks (RRs) for the association between vaginal-only versus abdominal/combined (reference) route of repair and failure to close the fistula at the 3-month follow-up visit

	Total repaired abdominally/both abdominally and vaginally included in the analysis	Total repaired vaginally included in the analysis	RR (95% CI)
Unmatched, unadjusted	57	1216	1.42 (1.11–1.81)*
Unmatched, adjusted for indication for abdominal repair	57	1201	1.72 (1.29–2.29)*
Unmatched, adjusted for indication for abdominal repair, surgeon experience in conducting complex repairs, mid-vaginal location, partial and complete urethral damage	56	1138	1.41 (1.05–1.88)*
Matched sample, reduced propensity score model	46	92	1.98 (1.27–3.07)*
Matched sample, expanded propensity score model	30	60	1.40 (0.77–2.56)

CI, confidence interval.

* *P* < 0.05.

### The effect of route of repair on fistula closure by indication for repair

In analyses stratified by indication, among women with fistula meeting indications for abdominal repair, women repaired vaginally had twice the risk of failure relative to those repaired abdominally (RR, 1.97; 95% CI, 1.03–3.79); effect estimation among women in whom an abdominal approach was not indicated was not possible because of sparse cell sizes (Table 5).

**Table 5:** Relative risks for the association between vaginal-only versus abdominal/combined (reference) route of repair and failure to close the fistula at the 3-month follow-up visit across levels of indication for abdominal repair in the unmatched sample

	Abdominal approach not indicated		Abdominal approach indicated	
		
	Closed, *n* (%)	Not closed, *n* (%)	Closed, *n* (%)	Not closed, *n* (%)
Vaginal-only route of repair	637 (84.48)	117 (15.52)	339 (75.62)	108 (24.38)
Abdominal/combined abdominal/vaginal	5 (100.00)	0 (00.00)	46 (88.46)	6 (11.54)
RR (95% CI)		1.97 (1.03–3.79)*	–	

CI, confidence interval.

* *P* < 0.05.

## Discussion

The route of fistula repair was influenced by a combination of patient and fistula characteristics, as well as facility-level factors. Not surprisingly, published indications for an abdominal route of repair appeared to influence the decision to undertake an abdominal route of repair, and location of the fistula in an area accessible through the vagina, such as a urethral or mid-vaginal location, was associated with a vaginal route of repair. Most women undergoing abdominal repair met the typical indications for an abdominal repair. Those women who did not may have exhibited other unmeasured characteristics which prompted the surgeon to undertake an abdominal repair, or may have been repaired abdominally as a matter of surgeon preference. However, the vast majority of women who met the indications for an abdominal route of repair were, in fact, repaired vaginally. Indeed, both site and surgeon experience in conducting complex repairs were highly predictive of the surgical approach used. Notably, surgeon experience in conducting complex repairs was inversely associated with the decision to undertake an abdominal repair. In a subanalysis (not shown), we evaluated whether more experienced surgeons were less likely to subjectively classify a repair to be ‘complex’, controlling for fistula prognosis. This did not appear to be the case. Thus, a more likely explanation is that more experienced surgeons are better able to access a range of fistulas vaginally. Finally, a parity greater than three was associated with an abdominal route of repair. The reasons for this are unclear, although this finding is consistent with that of Morhason-Bello et al.,^15^ who reported that women repaired abdominally had undergone a significantly greater number of deliveries than those repaired vaginally.

A vaginal route of repair was associated with an increased risk of failure to close the fistula, relative to the abdominal route of repair, after adjusting for other factors. This finding is surprising, as one might expect that fistulas repaired abdominally would be more complex cases, and therefore have a worse prognosis. Indeed, our results contradict those of Kriplani et al.,^9^ who found that a vaginal route of repair was protective against incontinence (defined as residual incontinence or failure to close the fistula). Our results must be interpreted with caution. First, it is possible that the types of fistula that are more likely to be repaired abdominally (i.e. ureteric, trigonal or supratrigonal) are, in fact, more likely to have a better repair prognosis than fistulas more likely to be repaired vaginally (i.e. urethral fistulas). In addition, it is possible that an abdominal route of repair is undertaken for those cases that surgeons deem more likely to be successfully repaired, given the potential risks and longer recovery time associated with abdominal surgery. Alternatively, it is possible that the abdominal route of repair is, in fact, beneficial where the fistula is difficult to access vaginally. Indeed, unadjusted stratified analyses suggested that the risk of failure among women repaired vaginally may be elevated for those women in whom an abdominal repair was indicated relative to those in whom an abdominal repair was not. However, there were few women who underwent abdominal repair when it was not indicated, resulting in potentially unstable estimates.

The practical implications of a potentially beneficial effect of an abdominal surgical approach are limited. An abdominal approach of repair is primarily performed under general anaesthesia; the use of general anaesthesia requires additional skill on the part of clinicians, is more expensive than the local anaesthetics used for a vaginal route of repair^15^ and may not be routinely available in low-resource settings. Other resources required for an abdominal repair, such as a wide range of sutures, may similarly be unavailable. Moreover, abdominal repairs have been found to be associated with increased blood loss,^14^,^15^ UTI^15^ and longer hospital stay relative to vaginal repairs.^14^ This more invasive procedure may also increase the risk of surgical site infection, especially in poorly resourced surgical settings. Further research evaluating which fistula characteristics indicate the need for an abdominal approach, and the effect of a vaginal route of repair across the substrata of women defined by fistula characteristics, is warranted.

Our study has several limitations. In this multi-country observational study, perioperative procedures were highly collinear within sites, and varied substantially across sites. In such a context, it is possible that, at one or more levels of confounding variables, no-one was observed at one or more levels of the exposure^28^; this is termed a violation of positivity^29^,^30^ or ‘off-support’^31^ data. The use of regression models in this context means that comparisons are based on very sparse or model-dependent data; although results from such analyses may be correct, they rely on ‘heroic modelling assumptions’.^32^ Propensity score methods can minimise violations of positivity, in that patients who do not match on the probability of exposure are excluded from data analysis. However, results obtained using multivariable modelling were similar to those obtained using propensity score matching, increasing our confidence that our findings were not solely based on statistical extrapolation. In addition, we conducted complete-case multivariate analyses, which may bias results if observations are not completely missing at random. With the exception of the variable representing provider experience in conducting complex repairs, we assumed that the few missing observations were missing completely at random. To address potential bias introduced by the exclusion of 5% of the sample for whom information on provider experience in conducting complex repairs was missing, we conducted a sensitivity analysis, imputing different values for this variable for the nine surgeons for whom it was unavailable. Our results remained unchanged, increasing our confidence that no bias was introduced by excluding these cases. Our measures may also have been subject to some misclassification: there is no accepted standard among fistula surgeons for measures such as degree of scarring and tissue loss; these measures were therefore based on surgeons’ subjective reports. However, we assume that any misclassification of exposure variables would be nondifferential, independent of other errors, and therefore would bias study results towards a null effect. Another limitation of this study is that the small number of repairs conducted via the abdominal route may have prohibited the detection of small, significant effects. In particular, we were underpowered to test the presence of effect modification; nonetheless, stratified analyses demonstrated trends in the directions anticipated. Finally, our results cannot be generalised beyond the clinical contexts in which our study took place. Nonetheless, we collected data from a heterogeneous sample of women across several countries and multiple study sites, increasing the external validity of our findings.

Despite its limitations, this study represents the only comprehensive evaluation of factors that influence the choice of route of repair for urinary fistula surgery. It is the largest collection of data assessing predictors of fistula repair outcomes to date, and the only study of this scale to systematically follow women after discharge from the facility in order to determine the long-term effects of the procedures studied on fistula repair outcomes. The provision of fistula care and treatment services in developing countries is fraught with many challenges. In a context which has limited human and infrastructural capacity to meet high demand for repair services, finding ways of providing services in a cost-effective manner, without compromising surgical outcomes and the overall health of the patient, is critical. Additional cohort studies that are adequately powered to test hypotheses of effect modification are warranted to confirm whether an abdominal route of repair is indeed beneficial for certain patient populations.
